# A host-centric morphological profiling approach to identify repurposed antiviral drugs

**DOI:** 10.1016/j.isci.2026.116673

**Published:** 2026-07-10

**Authors:** Elin Asp, Jonne Rietdijk, Marianna Tampere, Hanna Axelsson, Duncan Njenda, Swapnil Potdar, Adelinn Kalman, Polina Georgieva, Maris Lapins, Flavio Ballante, Alicia Soler, Martin de Kort, Tero Aittokallio, Andrea Zaliani, Maria Kuzikov, Philip Gribbon, Donald Lo, Jordi Carreras-Puigvert, Brinton Seashore-Ludlow, Ola Spjuth, Päivi Östling

**Affiliations:** 1 Department of Oncology and Pathology and Science for Life Laboratory, Karolinska Institutet, 171 76 Stockholm, Sweden; 2 Department of Pharmaceutical Biosciences and Science for Life Laboratory, Uppsala University, 75124 Uppsala, Sweden; 3 Chemical Biology Consortium Sweden (CBCS), Science for Life Laboratory, Department of Medical Biochemistry and Biophysics, Karolinska Institutet, 17176 Stockholm, Sweden; 4 Department of Microbiology, Tumor and Cell Biology, Karolinska Institutet, 17176 Stockholm, Sweden; 5 Institute for Molecular Medicine Finland (FIMM), HiLIFE, University of Helsinki, Helsinki, Finland; 6 European Infrastructure for Translational Medicine (EATRIS ERIC), Amsterdam, the Netherlands; 7 Institute of Cancer Research, Department of Cancer Genetics, Oslo University Hospital, Oslo, Norway; 8 Oslo Centre for Biostatistics and Epidemiology (OCBE), Faculty of Medicine, University of Oslo, Oslo, Norway; 9 Fraunhofer Institute for Translational Medicine and Pharmacology (ITMP), 22525 Hamburg, Germany

**Keywords:** host-directed antivirals, cell painting, drug repurposing, morphological profiling, SARS-CoV-2, pandemic preparedness

## Abstract

Antiviral drug discovery has traditionally targeted viral proteins, while host-directed strategies remain underexplored. We present a systematic drug repurposing strategy that uses morphological profiling to identify host-targeting antivirals. Using cell painting, we demonstrate that SARS-CoV-2 infection can be accurately determined from the morphological profile of virus-infected cells. Moreover, morphological features reveal how host cells respond to viral exposure, offering insights into antiviral activity, host-cell health, and putative mechanisms of action of the compounds. Screening 5,275 repurposable compounds, we identified candidates that reversed the infected phenotype, including ones not detected by conventional cytopathicity and antibody-based assays. After deprioritization of confounding phospholipidosis, we present 74 hit candidates, including unreported compounds targeting host processes implicated in viral infection. This adaptable and scalable platform is suited for diverse viruses and cell systems. We provide a resource of open-access screening data, images, and analysis pipelines to advance antiviral discovery and pandemic preparedness.

## Highlights

• Cell painting captures host phenotypes to predict antiviral activity
• Repurposing screen of 5,275 compounds with multiplexed phenotypic readouts
• Identification of 74 candidate host-directed antivirals
• Scalable, high-content platform across viruses and cell lines

## Graphical abstract

**Figure:**
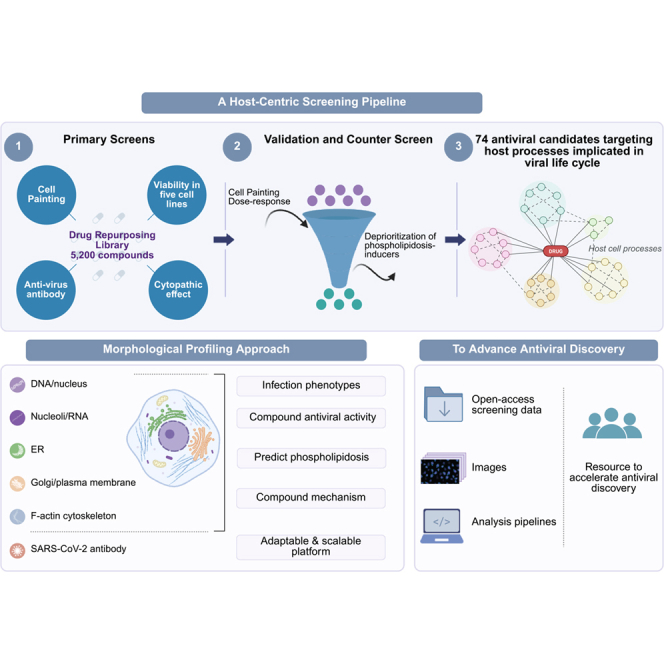

## Teaser

Virology; Drug delivery system; Medical imaging; Pharmacology; Cell biology; Computational bioinformatics

## Introduction

Over the past centuries, the emergence of pathogenic viruses, such as Ebola virus, Zika virus, and severe acute respiratory syndrome coronavirus 2 (SARS-CoV-2), has significantly impacted human health, resulting in hundreds of thousands of deaths annually.^1^^,^^2^^,^^3^ Several factors, including increased human and domestic growth, climate change, and global trade, have led to an increase in the number of viral outbreaks.^4^^,^^5^^,^^6^ Mutation-prone viruses—especially those with RNA genomes—evolve rapidly, increasing the risk of acquiring traits that enhance human infection or zoonotic spillover.^7^ In the event of a future pandemic, rapid methods for identifying effective antiviral agents against novel or re-emerging viral variants are vital.

Antiviral drugs can serve as a first line of defense during viral outbreaks, as they can be rapidly deployed, stockpiled in advance, and used when vaccines are unavailable or unsuitable for immunocompromised individuals.^8^^,^^9^^,^^10^ Small-molecule antivirals have already proven their value by reducing morbidity and mortality in infectious diseases, including hepatitis B, hepatitis C, HIV, and herpes simplex.^11^ There are two main classes of antiviral agents: direct-acting antivirals (DAAs) and host-directed antivirals (HDAs). While DAAs are most widely used, they are limited by the rapid emergence of resistance and typically lack broad-spectrum activity. In contrast, HDAs, which target host proteins involved in the virus life cycle, tend to offer broader-spectrum potential and a higher barrier to resistance, making them a promising strategy for rapidly mutating RNA viruses. Although inhibiting essential cellular functions can cause a greater risk of cellular toxicity.^12^^,^^13^^,^^14^

Morphological profiling has emerged as a powerful and cost-effective phenotypic screening approach in drug discovery. This image-based approach extracts thousands of single-cell features to generate high-dimensional morphological profiles and detect subtle changes in cellular organization. By capturing rich, multivariate data in a single assay, it has gained significant traction in drug discovery across applications, such as predicting compound mechanisms of action, assay activity, cell health, and disease-state reversal phenotypes across disorders, including Parkinson’s disease, Alzheimer’s disease, and cancer.^15^^,^^16^^,^^17^^,^^18^^,^^19^

Despite its broad adoption in drug discovery, its application in antiviral research remains relatively limited. High-content microscopy has been used to classify virus-infected cells without the need for an antibody marker, using high-throughput brightfield microscopy,^20^ fluorescent staining,^21^^,^^22^ and virtual staining approaches.^23^ More recently morphological profiling has been applied for antiviral drug discovery to identify drug repurposing candidates for COVID-19^21^ and to identify broad-spectrum antivirals with limited off-target effects.^24^ Unlike other phenotypic assays for antivirals, such as cytopathic effect (CPE) and antibody-based assays, morphological profiling can capture subtle cytopathic alterations well before cell swelling, fusion, or cell death, enabling interrogation of virus-cell interactions.^25^^,^^26^

Although these studies demonstrate the potential of morphological profiling for antiviral discovery, its implementation remains limited. The few studies that have applied this approach largely relied on a limited set of stains that detect viral infection along with nuclei, cytoplasm, and virus-focused markers or organelles, such as lysosomes and neutral lipids.^21^^,^^24^ To that end, we previously developed a cell painting-based approach to screen for antivirals, that provides in depth-insights into the host cell, and showed how this can be used to screen for both known and novel antiviral compounds.^27^

Here, we apply the cell painting assay, a widely used multiplexed untargeted staining method that labels eight key cellular compartments with fluorescent dyes and captures images across multiple channels to generate high-dimensional morphological profiles.^28^ We demonstrate that these rich profiles can be used to predict infection status based on antibody staining with high accuracy, identify virus-specific morphological signatures, and screen for compounds that reverse virus-induced phenotypes, provide insights into antiviral activity, host-cell health, compound confounders and putative mechanisms of action.

In this study, we present a systematic host-centered drug repurposing strategy using a diverse library of 5,275 compounds to identify antiviral candidates against SARS-CoV-2. We performed cell painting-based morphological profiling alongside conventional antiviral readouts, including CPE and antibody-based assays, enabling direct comparison across complementary assay modalities. We implemented a phospholipidosis counter screen to deprioritize confounding compounds. This integrated framework identified 74 candidate antivirals, including 44 not previously reported against SARS-CoV-2. Finally, we demonstrate that morphological profiling is generalizable across RNA viruses and cell types, cluster compounds sharing mechanism-of-action (MoA), and captures signatures associated with drug-induced phospholipidosis (DIPL).

We provide open access to a valuable collection of high-quality screening and high-content datasets generated across multiple assay platforms and cell lines. We believe this work offers a valuable framework and resource to accelerate the discovery of future host-directed antivirals.

## Results

### Morphological profiling captures a virus-induced phenotype and enables screening for antiviral compounds

To demonstrate the utility of morphological profiling for antiviral compound screening against SARS-CoV-2, we applied a previously developed combined cell painting and antibody-based assay to analyze subtle morphological changes in infected cells.^27^ Specifically, the staining panel targeted the nucleus, nucleoli, cytoplasmic RNA, endoplasmic reticulum (ER), Golgi apparatus, cytoskeleton, and plasma membrane. In addition to the cell painting stains, we incorporated an antibody-based viral stain to track viral infection levels. This assay enables the characterization of virus-induced morphological changes and screening for compounds capable of reversing the virus-induced phenotype.

**Figure 1:**
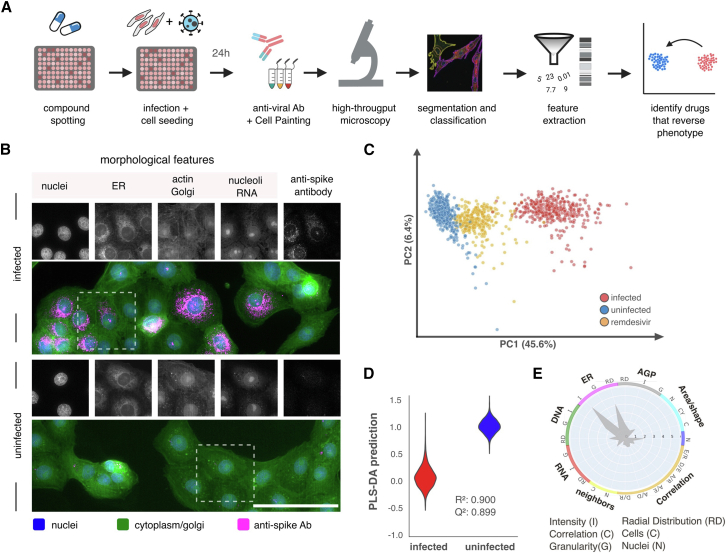
Morphological cell profiling workflow adapted for antiviral drug discovery (A) Schematic overview of the experimental workflow. Compounds are pre-spotted onto assay plates, followed by seeding of pre-infected cells. After 24 h of incubation, cells are fixed and stained with both a virus-specific antibody and cell painting dyes. This is followed by high-throughput imaging and feature extraction, enabling characterization of a virus induced phenotype and screening of compounds that could reverse the virus-induced phenotype. (B) Representative images of Vero E6 cells, either infected with SARS-CoV-2 or uninfected controls for each of the five imaging channels. Scale bars, 100 μm. (C) Principal-component analysis (PCA) applied to morphological features, with each point representing a single well. Percent variance explained by each component is indicated. (D) Observed versus predicted values from the PLS-DA (partial least squares discriminant analysis) model trained to classify infection status based on cell morphology features and corresponding model performance metrics. (E) Radar plot illustrating the most significantly changed morphological feature groups in response to viral infection compared to uninfected cells.

Briefly, the workflow involves seeding infected cells into multiwell plates containing the compounds. After 24 h, cells are fixed and stained with multiplexed immunofluorescence. High-throughput microscopy captures multiple imaging sites per well, after which images are processed using an image analysis pipeline that segments individual cells and extracts more than 1,500 morphological features per cell, including measurements of intensity, texture, and shape. These features are then aggregated and normalized to generate well-level morphological fingerprints, which are analyzed to identify compounds that mitigate or reverse the virus-induced phenotype (Figure 1A).

Figure 1B shows representative images of Vero E6 cells infected with SARS-CoV-2 across the five fluorescent channels. Approximately 70% of the cells are infected, as determined by the antibody staining. To assess whether infection induces detectable morphological changes, we performed principal-component analysis (PCA) on the extracted morphological features. As shown in Figure 1C, PCA revealed clear separation between infected, uninfected, and remdesivir-treated morphological profiles, indicating a distinct infection-induced phenotype that is partially rescued by antiviral treatment. Supervised classification using partial least squares discriminant analysis (PLS-DA) yielded R^2^ and Q^2^ values of 0.900 and 0.899, respectively, indicating strong separation between infected and uninfected samples based on morphological features alone (Figure 1D).

Next, we investigated whether cell painting features captured subtle phenotypic changes induced by SARS-CoV-2 infection. Infected cells exhibit pronounced changes across several organelles, including increased ER fluorescence intensity and altered radial distribution, as well as alterations in nuclear morphology and actin organization. These observations suggest that SARS-CoV-2 infection induces widespread remodeling of host-cell architecture. The most prominent effects are seen in the ER, consistent with virus-driven reorganization to facilitate replication (Figure 1E). This aligns with the images shown in Figure 1B, which displays increased ER brightness in infected cells.

#### Drug repurposing screens against SARS-CoV-2

To identify repurposable drug candidates against SARS-CoV-2, we systematically screened a library of 5,275 compounds using four complementary assays: a cell viability assay to assess compound toxicity, an antiviral rescue of CPE, virus-antibody staining, and morphological profiling. The compound library, originally curated by the Drug Repurposing Hub, comprises a chemically diverse set of small molecules that have reached clinical development stages, including approved, trial-phase, and withdrawn drugs. Most compounds have known mechanisms of action and target classes, including G-protein-coupled receptors, kinases, ion channels, nuclear hormone receptors, and voltage-gated sodium channels.^29^

### Cell viability assessment to select non-toxic concentrations

**Figure 2:**
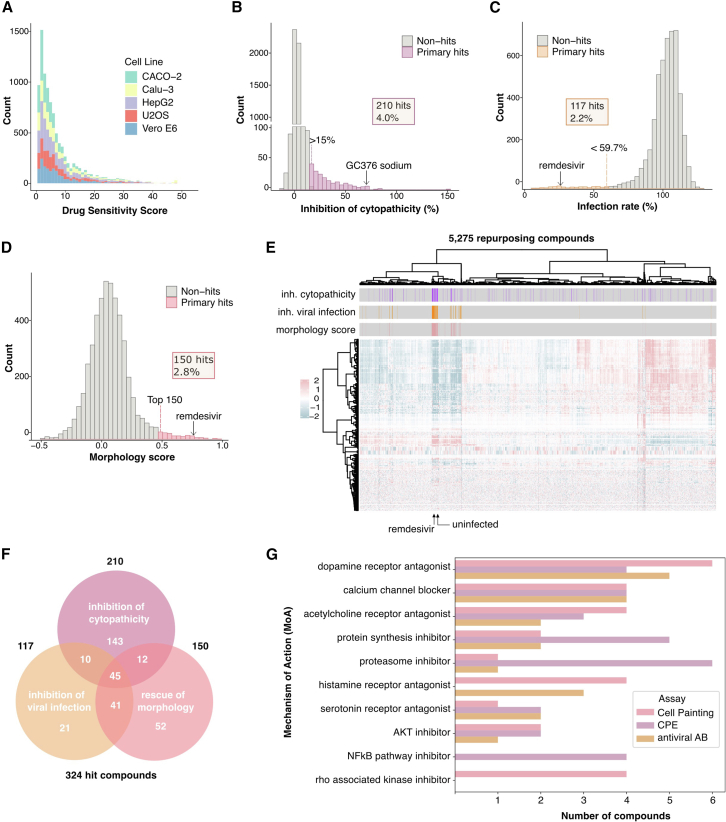
Screening of a drug repurposing library of 5,275 compounds using four assay readouts (A) Overview of viability screening across five cell lines, used to calculate sensitivity scores and inform concentration selection for follow-up assays. (B–E) Histograms showing the distribution of assay values for (B) cytopathicity inhibition (best dose selected per compound), (C) viral infection rate, and (D) morphology scores. Thresholds used to define hits are indicated. (E) Unsupervised hierarchical clustering of morphological profiles for all 5,275 compounds using Ward’s linkage method and the Euclidean distance metric; on top of the heatmap we show the hits for the three respective readouts. Inh, inhibition. (F) Venn diagram depicting the overlap of compounds identified as hits across the three assay readouts. (G) Bar chart summarizing the number of compounds corresponding to the top 10 mechanisms of action, with counts shown per assay type. See also Figure S1.

When screening large and chemically diverse compound libraries, differences in compound potency can cause active compounds to be missed if only a single concentration is tested, particularly when cytotoxicity masks antiviral effects. To address this, we first performed a viability screen to assess compound toxicity. Viability was evaluated across five cell lines using the drug sensitivity score (DSS), allowing a generalizable assessment of cytotoxic effects (Figure 2A, Tables S1, S2, S3, S4, and S5). Overall, the majority of the compounds in the drug repurposing set caused no major signs of toxicity across the cell lines, with an average DSS of 1.3. HepG2 and Calu-3 cells exhibited slightly higher compound toxicity, with 5.7% and 4.9% of compounds with a DSS > 10, respectively. Based on the viability screen, the highest non-toxic concentration, either 0.83 or 10 μM, with > 80% viability in Vero E6 cells was selected for further experimentation. This concentration also guided dose selection for the subsequent validation screens, using a range spanning below and above the hit concentration.

### CPE assay identifies compounds preventing virus-induced cell death

In the CPE assay, compounds were evaluated for their ability to prevent virus-induced cell death, serving as a functional readout of phenotypic antiviral activity. The full compound library was screened across five concentrations from 1 nM to 10 μM. A small subset of compounds reduced virus-induced cytopathicity (Figure 2B, Table S6). We selected compounds that, at one or more tested concentrations, mitigated the virus-induced reduction in cell viability by 15% or more, resulting in a set of 210 candidate compounds.

### Antibody-based detection of SARS-CoV-2 reveals compounds reducing infection rate

Antibody detection provides a direct approach for identifying compounds that reduce viral infectivity. Here, we used the antibody channel, which was included in the morphological profiling assay, to identify compounds that lowered viral infection levels. Compounds that reduced infection by more than 40% were selected, yielding 117 candidate compounds (Figure 2C, Table S7).

### Morphological profiling highlights hits reversing the virus-induced phenotype

To identify compounds capable of reversing the virus-induced morphological phenotype, we introduce a morphology score that integrates morphological features from all stained organelles into a single metric, quantifying the similarity of each cellular state to that of uninfected, healthy cells. Compounds with higher scores exhibited profiles more closely resembling non-infected controls. Based on this metric, we prioritized the top 150 compounds for follow-up experimentation (Figure 2D, Table S7).

To further explore phenotypic similarities among these hits, we applied hierarchical clustering with Ward’s linkage and Euclidean distance. This analysis revealed a cluster of compounds that shared similarity in terms of their morphology to the non-infected controls. This cluster was enriched for compounds that reduced the number of infected cells and inhibited CPEs (Figure 2E).

Based on the results from these three primary screening read-outs, we selected the hits from each of read-outs for further validation. In total, 324 compounds were prioritized for follow-up testing. Among these, there was an overlap of 57 compounds between the cell painting and CPE assay, 86 compounds between the cell painting and antibody-based screen, and 55 compounds between the antibody-based screens and the CPE assay. Forty-five compounds were identified as hits across all three assay types (Figure 2F). Morphology score correlated strongly with viral infectivity (r = − 0.78), demonstrating that compounds reducing infection consistently rescued cellular morphology toward an uninfected phenotype. Cytopathicity showed markedly weaker associations with both morphology (r = 0.21) and infectivity (r = − 0.37), with high dispersion. This limited correlation may reflect differences in assay sensitivity and readout time point or indicate that cytopathicity captures a partly independent dimension of antiviral activity not fully reflected by infectivity or morphology (see Figure S1A).

The three assays identified compounds with diverse MoAs, covering a total of 204 distinct mechanisms. Figure 2G shows the top ten MoAs among the hit compounds, along with the number of compounds identified per assay for each MoA. The most prevalent MoA among the hit compounds was dopamine receptor antagonists, which was identified in all three assays. Notably, morphological analysis uniquely identified Rho-associated kinase (ROCK) inhibitors as hits that were not detected in the other two readouts. Conversely, inhibitors of the NF-κB pathway were exclusively identified in the CPE assay.

#### Antiviral activity validated in human lung epithelial A549^ACE2^ cells and assessment of drug-induced phospholipidosis

**Figure 3:**
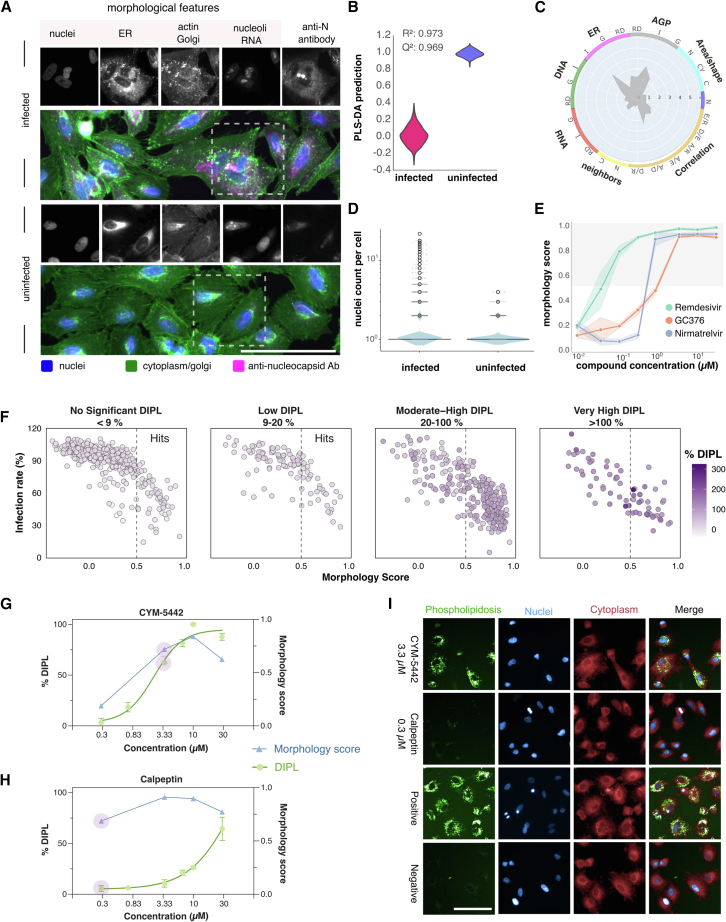
Validation and deprioritization of hit compounds in human lung epithelial cells using morphological profiling and DIPL assessment (A) Representative cell painting and immunostaining images of infected and non-infected A549^ACE2^ control wells showing distinct morphological changes upon SARS-CoV-2 infection. Scale bars, 100 μm. (B) Observed versus predicted values from the PLS-DA model trained to classify infection status based on cell morphology features and model performance metrics. (C) Radar plot illustrating the most significantly changed morphological feature groups in response to viral infection. (D) Nuclei count per cell of infected and uninfected cells indicating syncytia formation of infected cells. (E) Dose-response plot of antiviral control compounds based on morphology scores. Shaded regions show the 95% confidence interval. The gray area highlights the dose range where compounds successfully reversed the virus-induced phenotype. (F) Faceted scatterplots showing the relationship between morphology score (*x* axis) and SARS-CoV-2 infection rate (*y* axis), stratified by drug-induced phospholipidosis levels (% DIPL). Each panel represents a DIPL category and each point represents a compound-concentration pair from the 324 hit compounds, colored by % DIPL intensity. The vertical dashed line indicates the morphology score threshold (>0.5). Selected hits fall within the range ≤20% DIPL and morphology score >0.5. (G and H) Dose-response curves of CYM-5442 and calpeptin, showing %DIPL (left *y* axis, green color) and PLS-correlation (right *y* axis, blue color), across log-transformed compound concentrations (*x* axis). The lowest concentration reaching the morphology score threshold (>0.5) is annotated in purple. DIPL data points are represented by the mean ± SD of two replicates and dose-response curves were fitted using a four-parameter model in GraphPad Prism (v.10.5.9). (I) Representative fluorescence images of uninfected A549^ACE2^ cells treated with CYM-5442 (3.3 μM), calpeptin (0.3 μM), positive control (tamoxifen), and negative control (DMSO), at 24 h. Stains: phospholipidosis (green), nuclei (blue), cytoplasm (red). Scale bars, 100 μm. See also Figure S1. See also Figure S2.

To validate the compounds identified in the three primary screens, we tested the 324 hit compounds in a four-point dose-response assay (0.1–3.3 μM or 0.3–30 μM) in human A549^ACE2^ cells using morphological profiling. To determine whether SARS-CoV-2 infection induces a detectable morphological phenotype in A549^ACE2^ cells, we first evaluated the response of this cell model to infection. As shown in Figure 3A, representative images of infected and non-infected control wells revealed clear morphological changes across the different channels. Using PLS-DA, we found that morphological features alone could perfectly separate infected from non-infected conditions, achieving an area under the ROC curve (AUC) of 1.000, and a *R*^2^ and *Q*^2^ score of 0.973 and 0.969, respectively (Figure 3B).

Similar to the effects observed in Vero E6 cells, phenotypic feature groups that showed significant changes upon infection included ER intensity and radial distribution, cytoskeletal organization, and Golgi (AGP) staining, as well as correlation-based features. Moreover, the images revealed a pronounced increase in nuclei per cell and large multinucleated cells, indicative of syncytia formation^30^ (Figures 3A and 3D).

A total of 208 (64.8%) compounds exhibited the ability to reverse the virus-induced phenotype (morphology score > 0.5). We observed dose-dependent rescue of cellular morphology following treatment of effective antiviral compounds (Figure 3E, Table S8). While responses varied between the two cell lines, and the correlation of morphology scores in Vero E6 and A549^ACE2^ cells was modest (See Figure S1B), 81 (25%) of the tested compounds achieved a morphology score above 0.5 in both cell lines, indicating partial to substantial reversal of the virus-induced phenotype (See Figure S1C, Table S8).

To prioritize antiviral candidates, we integrated the morphology scores, infection rates and DIPL responses (Figure 3F). Compounds were classified as no significant DIPL ( < 9%), low (9%–20%), moderate-high (20% –100%), and very high ( > 100%). A trend of decreased infection rate with increasing DIPL levels was observed, especially beyond 20% DIPL (Figure 3E; Figure S2A). Based on this, a threshold of deprioritizing compounds with > 20% DIPL was set. A total of 74 compounds had a morphological score (> 0.5) and no to moderate DIPL levels ( ≤ 20%). The selection process is further visualized in Figure S2B. In Figures 3G and 3H, we illustrate the relationship between the DIPL score and the morphology score for a deprioritized compound, as well as a prioritized hit compound. While both compounds demonstrate a phenotypic rescue (morphology score > 0.5), CYM-5442 exceeds the DIPL threshold at its active antiviral concentration and was therefore deprioritized (Figure 3G). In contrast, calpeptin induced morphological rescue without exceeding DIPL limits at its lowest effective antiviral dose (Figure 3H). Representative immunofluorescence images show the phospholipid accumulation of both compounds and controls (Figure 3I).

In addition, a clear dose-dependency in phospholipid accumulation was observed. At 0.3 and 0.8 μM, most compounds (89% and 73%) exhibited no significant DIPL, while concentrations from 3.3 μM and above showed higher levels of DIPL; at a concentration of 30 μM, 50% of the compounds induced moderate-high DIPL (Table 1; Table S9). Moreover, we evaluated the DIPL development across three time points (24, 48, and 72 h) and observed that EC_50_ values were lowest at 24 h, indicating the most sensitive time point for DIPL detection, with EC_50_ values reached at lower concentrations (see Figure S2C, Table S9). For this study, the 24 h time point was evaluated to align with the time of infection and compound treatment. Additionally, five impure compounds and previously reported DIPL inducers not active in our screen due to low solubility or cell line specificity were excluded from further analysis. The most common MoAs among the DIPL inducers were dopamine receptor antagonists (*n* = 7), followed by calcium channel blockers (*n* = 5), serotonin receptor antagonists (*n* = 4), and histamine receptor antagonists (*n* = 4) (Figure S2D).

**Table 1:** Distribution of compounds across concentrations and drug-induced phospholipidosis categories

DIPL category/Concentration (μM)	0.3	0.8	3.3	6.6	10	30
No significant DIPL (<9%)	89.4%	73.2%	45.1%	34.4%	31.2%	23.9%
Low DIPL (9–20%)	8.4%	16.3%	12.8%	10.7%	9.2%	9.0%
Moderate-High DIPL (20–100%)	1.6%	10.2%	37.5%	41.6%	41.4%	50.2%
Very High DIPL (>100%)	0.6%	0.3%	4.6%	13.5%	18.2%	16.9%

DIPL, drug-induced phospholipidosis.

#### Integrated antiviral screening approach reveals unreported antiviral candidates and insight into key host processes

Our integrated screening framework identified 74 prioritized hits. Based on available literature and PubChem records, 44 (59%) of these compounds have not been previously reported as SARS-CoV-2 inhibitors (Table S10). Most hits demonstrated activity in the low micromolar range, with 31 compounds (42%) active at 3 μM, 21 compounds (28%) active at 0.1–1 μM, while only five (7%) required 30 μM (Table 2). Overall, the hits exhibited robust rescue of infection-induced morphological phenotypes. The strongest rescuing effects on host-cell morphology were observed for the p38 MAPK inhibitor LY2228820, the CDK inhibitor BS-181, and the calpain inhibitor calpeptin.

**Table 2:** Minimal effective concentration of validated 74 antiviral candidates

Minimal effective concentration (μM)	Number of compounds (*n*, %)
0.1–1 μM	21 (28%)
3 μM	31 (42%)
10 μM	17 (23%)
30 μM	5 (7%)

**Figure 4:**
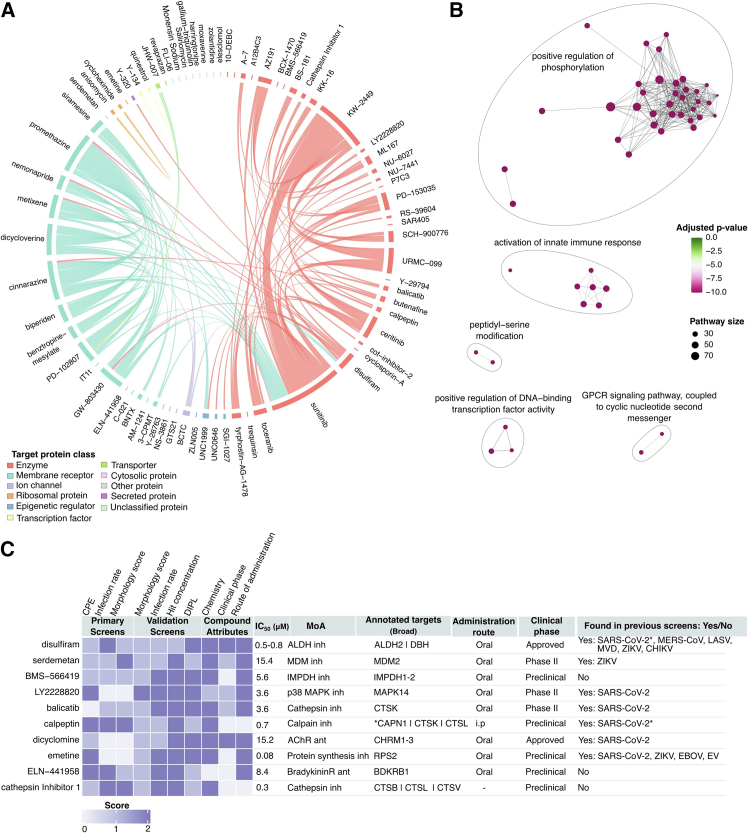
Host biological pathways across the protein targets and prioritization of phenotypic hits (A) Chord diagram of shared host targets among the prioritized 74 compounds. Chords indicate shared host targets between compounds, with thickness proportional to the number of shared targets. Sector width reflects total number of shared targets per compound. Square-root scaling was applied. (B) Gene Ontology enrichment analysis on the 700 annotated targets visualized by the top 50 enriched terms (adjusted *p* value <0.05). Nodes represent the enriched GO Biological Process pathways and edges represent the similarity between the pathways based on shared gene sets. Node size reflects the number of genes associated with each pathway, and node color indicates pathway significance by adjusted *p* value. (C) Target product profile hit scoring system. A heatmap of the top 10 ranked repurposable compounds based on combined scores from the primary screens, validation screens, and compound attributes. The chemistry category includes molecular weight (MW) and logP value. MW ≤ 500 and logP≤5 was ranked highest. The hits were given points 0–2 in each category and were summarized for ranking and listed in descending order with representative IC_50_ values recorded from the dose-response analysis. Additional metadata includes MoA, primary annotated targets from the Clue.io Repurposing Hub, highest clinical phase, administration route, and reported SARS-CoV-2 and other viral activity based on literature and PubChem bioassay records. Oral∗ indicates the compound is only used experimentally. SARS-CoV-2∗ indicates activity against viral proteins. Calpeptin targets are sourced from REMEDi4ALL annotations. CPE, cytopathic effect assay, DIPL, drug-induced phospholipidosis, inh, inhibitor, ind, inducer, ant, antagonist, R, receptor, .p, intraperitoneal, i.v, intravenous, MERS-CoV, Middle East respiratory syndrome-related coronavirus, SARS-CoV, severe acute respiratory syndrome coronavirus, LASV, lassa virus, MVD, marburg virus, ZIKV, zika virus, CHIKV, chikungunya virus, EBOV, ebola virus, EV, enterovirus. See also Figure S3.

To gain biological insights into the common drug targets of our hits, we evaluated the 700 annotated targets associated with the 74 hits, obtained from Clue.io Repurposing Hub and REMEDi4ALL.^29^^,^^31^ These spanned across nine major protein classes, with kinases and G protein-coupled receptor (GPCRs) and as the most represented subclasses (Figure 4A). Notably, 45 compounds shared 192 targets, indicating substantial target overlap. We compared our dataset to a CRISPR-knockout screen of SARS-COV-2 host factors, revealing that 43 of our targets overlapped with essential host factors identified in the screen, representing hits across 21 compounds.^32^

Gene Ontology enrichment analysis revealed five overrepresented biological processes: positive regulation of phosphorylation, activation of innate immune response, GPCR signaling, peptidyl-serine modifications, and positive regulation of DNA-binding transcription factor activity (Figure 4B). These clusters highlight host signaling and transcriptional regulatory pathways known to be exploited by SARS-CoV-2.^33^^,^^34^^,^^35^^,^^36^^,^^37^

To prioritize compounds with translational potential, we applied a target product profile (TPP)-based^38^ scoring system integrating performance across the antiviral screens, chemical properties, clinical developmental phase, and route of administration. By requiring convergence across these independent readouts, capturing viral protein levels, host cell responses, and cell viability, we enrich compounds with consistent antiviral activity. The top ten prioritized candidates are shown in Figure 4C (full list of 74 compounds in Table S10), which include eight orally formulated candidates of which four have not been identified in prior SARS-CoV-2 screens. Three of the compounds have shown activity across multiple viruses, according to literature and PubChem bioassay records. Notably, the prioritized subset retained a prominent mechanistic cluster of three cathepsin inhibitors.

To further confirm antiviral activity of the top ten compounds, we performed dose-response validation using antibody-based detection of SARS-CoV-2 nucleocapsid protein (Figure S3). All ten compounds showed dose-dependent reduction in viral protein levels of which four compounds exhibited sub-micromolar IC_50_ values, three showed low micromolar potency, and three were active in the high micromolar range.

Collectively, these findings demonstrate that our integrated screening framework identifies both unreported host-targeting candidates and previously recognized antiviral agents acting on shared biological processes.

#### Morphological profiling provides rich profiles and enables detection of compound-induced cellular effects and mechanistic signatures

**Figure 5:**
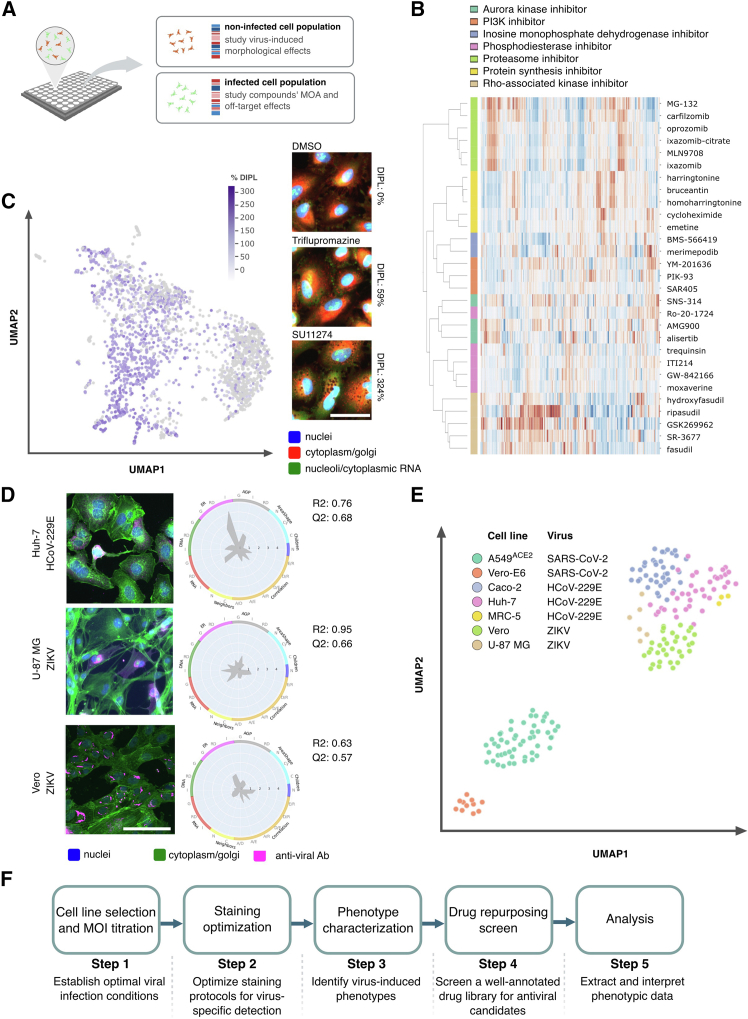
Profiling of infected and non-infected cells reveals virus-specific phenotypes and compound effects (A) Schematic overview of the data-splitting strategy: morphological profiles were separated into infected and non-infected cell subsets to enable independent analysis of virus-induced phenotypes and compound-induced mechanisms of action. (B) Unsupervised hierarchical clustering of compounds based on morphological profiles of non-infected cells, grouped by their annotated mechanisms of action (MoA), using cosine similarity and complete linkage. (C) UMAP projection of non-infected cell profiles colored by phospholipidosis levels as measured by the DIPL assay. Representative images of selected compounds show characteristic vacuolization for DIPL inducing compounds (blue: nuclei, fuchsia: endoplasmic reticulum, green: nucleoli, cytoplasmic RNA). Scale bars, 50 μm. (D) Representative images and radar plots for virus-infected cells in different cell lines (Huh7 with HCoV-229E, U-87 MG with ZIKV, and Vero with ZIKV). Strength and reproducibility of the profiles are indicated by the R^2^ and Q^2^ values. Fluorescent images labeling used in imaging: nuclei (blue), virus-specific antibody (fuchsia), and a cytoplasmic marker including cytoskeleton, Golgi, and plasma membrane (green). Scale bars, 100 μm. (E) UMAP visualization of morphological profiles of infected cells across different cell lines and viruses. (F) Workflow for rapid morphological profiling in response to emerging viral outbreaks and pandemic preparedness. See also Figure S4.

To leverage the information-rich morphological profiles, we explored their potential to capture compound-induced cellular effects and mechanistic insights into compound activity. Using single-cell resolution of our assay, we separated non-infected and infected populations of the treated A549^ACE2^ cells and aggregated profiles to create perturbation level morphological profiles (Figure 5A). We first evaluated how many compounds resulted in significant morphological effects by calculating mean average precision (mAP) scores at the perturbation level (defined as each unique compound-concentration combination) for non-infected cells. Here, phenotypically active refers to perturbations that produce profiles distinguishable from negative controls. We used cosine similarity to rank all profiles by similarity and determined the statistical significance of the mAP scores using permutation testing.^39^ In total, 93% of the compounds exhibited statistically significant (*p* < 0.05 for corrected *p* value) and reproducible morphological effects for at least one of the tested doses. Across all conditions in the screen, 58% of the perturbations induced a significant morphological effect in the uninfected cell populations (see Figure S4A).

To examine whether the morphological profiles could reveal mechanistic insights, we tested if compounds with the same MoA produced similar phenotypic signatures. Using mAP, we selected phenotypically consistent MoAs represented by two or more compounds. Unsupervised hierarchical clustering of compounds from seven selected MoAs, showed that compounds with the same mechanism had similar profiles and clustered together (Figure 5B). Among the compound groups, proteasome inhibitors formed the most distinct cluster, characterized by highly consistent morphological profiles evident in the heatmap. Except for Ro-20-1724, all compounds clustered with other compounds sharing the same MoA.

Next, we assessed whether compounds that induce DIPL exhibit distinct morphological profiles. Using the non-infected cell profiles, we generated a uniform manifold approximation and projection (UMAP) embedding of all conditions with matched doses that had been tested in the phospholipidosis assay (Figure 5C), coloring each point according to the assay results. This revealed that most of the phospholipidosis-inducing and non-inducing compounds separated into distinct morphological clusters. Representative images show phospholipid accumulation visible in cell painting images as small dark vesicles in the cytoplasm, increasing with greater levels of DIPL.

To further quantify these effects, we calculated the Pearson correlation of all morphological profiles to fluoxetine hydrochloride, a well-characterized phospholipidosis inducer included in the assay. Notably, all compounds with high similarity to fluoxetine hydrochloride (Pearson correlation between 0.7 and 1.0) were identified as moderate or strong DIPL inducers (defined as >20% induction; see Figure S4B). A correlation matrix of all compounds (see Figure S4C) confirms that most DIPL-inducing compounds exhibit high similarity to one another, including other well-known cationic amphiphilic drugs (e.g., prochlorperazine, triflupromazine). However, not all DIPL-inducing compounds were highly correlated to each other; several compounds with moderate to high DIPL levels showed lower similarity, possibly due to additional compound-induced effects beyond phospholipidosis.

#### Virus-specific morphological signatures detected across diverse cell models

To evaluate the broad applicability of our approach across different cell models and viral infections, we profiled three viruses, human coronavirus 229E (HCoV-229E), Zika virus (ZIKV), and SARS-CoV-2, each with two to three permissive cell lines. For every condition, we analyzed the morphological profiles of infected cell populations within each well and normalized them against non-infected cells to reduce well-level confounding effects (Figures 5D and S4D). A UMAP visualization of the resulting morphological profiles (Figure 5E) shows that each virus elicits a distinct and reproducible phenotypic signature. Infected cells tend to cluster according to the infecting virus, regardless of the cell line used, highlighting that virus-specific morphological signatures are conserved across cellular models.

The strength of these morphological effects varies both between viruses and across cell lines, as indicated by PLS metrics. Among the viruses tested, SARS-CoV-2 consistently induces the most pronounced morphological effects, followed by HCoV-229E and lastly ZIKV. Notably, HCoV-229E-infected cells exhibit specific changes in ER morphology, similar to those observed in SARS-CoV-2 infection. In contrast, ZIKV infection results in a distinct morphological signature characterized by various affected correlation features as well as effects on neighboring nuclei.

#### Standardized screening workflow for repurposed antivirals

To facilitate rapid screening in the context of emerging viral outbreaks and for pandemic preparedness, we developed guidelines for applying morphological profiling to new cell models and viruses. The workflow consists of five steps: (1) cell line and multiplicity of infection (MOI) optimization, (2) staining optimization, (3) phenotype characterization, (4) drug repurposing library screening, and (5) data analysis (Figure 5F).

*Step (1) Cell line selection and MOI titration*: select a permissive cell line that forms monolayers in multiwell assay plates. Use an MOI that yields ∼50%–70% infection rate while maintaining cell viability. Maintaining both infected and uninfected populations enables analysis of infection-associated phenotypes alongside uninfected cell phenotypes such as MoA-clusters and DIPL. Higher infection rates and later time points might cause excessive cell death, confounding morphological profiles associated with cytotoxicity and limiting the ability to analyze uninfected populations.

*Step (2) Staining optimization*: incorporate a virus-specific antibody to monitor infection and infection levels across plates. Because cell painting uses all five fluorescence channels, we replaced MitoTracker to accommodate the antibody, as it requires live-cell staining and is impractical in BSL-3/4 conditions. To minimize spectral overlap, place the antibody in the highest wavelength channel (e.g., Cy5/Alexa 647) to compensate for the weaker signals than the dyes. Finally, optimize the washing steps to protect cell morphology and image quality to prevent interference with downstream morphological analysis.

*Step (3) Phenotype characterization*: to rapidly extract morphological features from images, we share an automated CellProfiler pipeline that integrates Cellpose for robust cell segmentation, enabling high-throughput and consistent analysis across experimental conditions. By separately identifying nuclei and cytoplasm, it effectively accommodates highly multinucleated cells typical for some viral infections. The extracted features can then be analyzed using the provided Jupyter notebooks to compare infected and non-infected conditions and identify a screenable phenotype. Use PLS-DA to determine the morphological effects of infection and guide the final selection of the cell line and MOI for downstream compound screening.

*Step (4) Compound library and screening setup*: incorporate sufficient uninfected and infected DMSO-treated wells and known antivirals to benchmark assay performance. Randomize the controls and compounds by using tools such as PLAID to minimize position bias.^40^ Consider including assay interference compounds, such as phospholipidosis inducers and conduct a DIPL counter-screen if phospholipidosis is a confounding factor to the observed antiviral activity.

*Step (5) Analysis*: Use the provided Jupyter Notebooks to compute the morphology scores and prioritize compounds that reverse virus-induced phenotypes. Target enrichment analysis can be performed by linking phenotypic responses to known compound targets, aiding in the biological interpretation and prioritization of candidate hits.

Running the assay requires: access to a biosafety laboratory appropriate for the viral pathogen’s containment level, high-throughput fluorescent microscope, adequate computational power and storage and CellProfiler installations for parallelized image analysis. We refer to the original cell painting guidance provided in Bray et al., and Cimini et al., for detailed protocols on automated image acquisition, computational setup, and laboratory equipment requirements.^28^^,^^41^

## Discussion

Host-targeting antiviral strategies offer a promising approach for addressing emerging viral threats, although identifying compounds that effectively modulate host pathways remains challenging with current antiviral approaches. In this study, we present a systematic drug repurposing strategy that addresses this challenge by employing morphological profiling to identify host-targeting antiviral candidates.

Phenotypic antiviral screens are often CPE-based assays, that rely on indirect, endpoint-based readouts of viral replication and therefore provide limited resolution of early or subtle cellular phenotypic effects. As these assays typically detect virus-induced cell death at later stages of infection (48–72 hpi) and are limited to lytic viruses, they may overlook partial antiviral effects and are be prone to both false positive and false negative signals, such as proliferative effects independent of viral infection.^42^^,^^43^^,^^44^^,^^45^^,^^46^ In contrast, our adapted cell painting assay can detect phenotypic changes at earlier time points (24 hpi) and subtle infection-associated effects. By integrating a virus-specific antibody, we enable simultaneous single-cell quantification of viral infection and high-dimensional morphological profiling of host cell responses.

Our results demonstrate that SARS-CoV-2 infection induces a characteristic and robust morphological phenotype in both Vero E6 and human A549^ACE2^ cells, with morphological features alone achieving near-perfect separation between infected and uninfected cells. In addition, cell painting captured virus-induced cellular effects, including pronounced ER changes and syncytia formation, consistent with known effects of SARS-CoV-2 replication^30^ which is associated with COVID-19 lung pathology.^47^

While previous studies have demonstrated that morphological profiling can classify infection states^24^^,^^48^^,^^49^ and have applied this approach for antiviral screening and compound triaging,^24^^,^^25^ our work extends these efforts by leveraging an untargeted cell painting panel across multiple organelles, enabling deeper characterization of host-cell responses. We further demonstrate that the morphological features capture signals associated with compound mechanisms of action and confounding effects such as drug-induced phospholipidosis, highlighting the potential of cell painting as an approach for detecting these effects without requiring a dedicated counter-screen. In addition, we directly benchmark morphological profiling against CPE and antibody-based readouts within the same experimental framework.^47^

Comparison of primary screening results revealed substantial overlap between cell painting and antibody-based assays, which were highly correlated, whereas the CPE assay yielded a distinct group of candidate compounds. This likely reflects fundamental differences in readout strategy, capturing distinct stages and aspects of viral infection. The antibody-based assay directly quantifies viral proteins, the CPE assay indirectly measures antiviral activity, and cell painting captures the global complexity of the host cell responses in an unbiased, high-dimensional manner. Despite limited overlap at the compound level, hits across all assays converged similar mechanisms. Notably, dopamine receptor antagonists were the most frequent MoA identified in the primary Vero E6-based screens; however, this class was also strongly enriched among DIPL-inducers. Importantly, following the counter screen, only one dopamine receptor antagonist remained, indicating that the antiviral activity could be a reflection of an unwanted DIPL artifact.

Following triaging, 74 compounds remained that reverted the virus-induced phenotype without inducing significant DIPL. These candidates converged on host pathways commonly hijacked by viruses, with kinase signaling emerging as a prominent mechanistic class, with the p38 MAPK inhibitor LY2228820 emerging among the high-ranking hits. Several studies show that viruses modulate kinase signaling for immune evasion, cell-cycle regulation, and cytoskeleton reorganization, providing a plausible mechanistic basis for the enrichment of kinase-modulating compounds observed.^37^^,^^50^^,^^51^^,^^52^

Notably, 43 targets overlapped with essential SARS-CoV-2 host factors identified in a CRISPR screen by Daniloski et al.^32^ Among these, PPID and PIK3C3 were highlighted as targets of particular interest. PPID, targeted by cyclosporin A, was identified as an essential protein acting in ER-golgi trafficking. PIK3C3, targeted by the compound SAR405 and involved in endosomal trafficking, was highlighted as a promising drug target.^32^^,^^53^ While a study demonstrated that SAR405 was not effective *in vivo* due to pharmacokinetic limitations, modeling approaches improved these properties and demonstrated strong anti-coronaviral efficacy.^53^

In addition, we identified three cathepsin L-targeting compounds, in which the target has been shown to facilitate SARS-CoV-2 entry via the spike protein, and inhibition blocks infection *in vitro* and *in vivo.*^54^ Interestingly, eight muscarinic AchR antagonists were identified. Although muscarinic AchRs have not been directly linked to SARS-CoV-2, GPCR signaling and membrane trafficking has been suggested to play a role in viral infection and pathogenesis and warrants further investigation.^36^^,^^55^^,^^56^ Overall, 44 compounds represent, to our knowledge, previously unreported candidates against SARS-CoV-2.

In summary, we present a scalable and information-rich antiviral screening strategy that captures antiviral effects, cellular phenotypes, and mechanistic insights within a single assay. We screened a drug repurposing library and identified compounds with previously reported multi-virus antiviral activity, alongside new candidates, and demonstrated applicability across diverse viruses and cell systems. We provide open access to an extensive collection of high-quality screening datasets for 5,275 drugs in the repurposed library to support future antiviral discovery and outbreak preparedness.

### Limitations of the study

One limitation of our approach is the reliance on cell lines that, while easy and permissive to the virus, may not fully capture the complexity of human airway biology. Vero E6 cells, for example, are of non-human origin, and lack functional interferon signaling, which may limit the relevance of host-targeting antiviral effects.^57^ Although we validated hits in human A549^ACE2^ cells, future studies would benefit from using more physiologically relevant models, such as primary bronchial epithelial cells.^58^

Additionally, several compounds show antiviral activity at concentrations exceeding their annotated target IC_50_ values, raising the possibility of unspecific or off target mechanisms. Genetic validation, such as siRNA or CRISPR knockdown of the proposed targets would strengthen them as targets for antiviral activity. Future implementations could also adapt the staining panel to expand the biological coverage, for instance, including a mitochondrial dye, given this organelle is known to be affected by viral infection,^59^ or tailor the assay toward specific host-cell responses or toxicity endpoints.

## Resource availability

### Lead contact

Requests for further information and resources should be directed to and will be fulfilled by the lead contact, Elin Asp (elin.asp@ki.se).

### Materials availability

This study did not generate new unique materials.

### Data and code availability

The datasets and computer code produced in this study are available at:

• Data are available in supplemental information data.
• Code for computing morphology to uninfected score, image analysis pipelines and extracted morphological features: https://github.com/pharmbio/sarscov2-phenomics-repurposing
• Cellular images are deposited at BioImage Archive^60^: https://www.ebi.ac.uk/biostudies/bioimages/studies/S-BIAD2580; accession number: S-BIAD2580.
• Any additional information required to reanalyze the data reported in this article is available from the lead contact upon request.

## Acknowledgments

This work was supported by the REMEDi4ALL consortium, which has received funding from the European Union’s 10.13039/100010661Horizon Europe research and innovation program under grant agreement no. 101057442. Views and opinions expressed are those of the authors only and do not necessarily reflect those of the European Union, who cannot be held responsible for them. This project additionally received funding within the SciLifeLab National COVID-19 Research Program and 10.13039/501100004063Knut och Alice Wallenbergs Stiftelse (KAW) (KAW 2020.0182; KAW 2020–0182, 2020-0032; KAW 2020.0241; V-2020-0699). O.S. acknowledges funding from the 10.13039/501100004359Swedish Research Council (grants 2020-03731, 2020-01865, 2024-04576, and 2024-03566), 10.13039/501100001862Formas (grant 2022-00940), Swedish Cancer Foundation (22 2412 Pj 03 H), and the Swedish strategic research programme eSSENCE. The authors acknowledge the support from the Chemical Biology Consortium Sweden (CBCS), 10.13039/501100007051Uppsala University and 10.13039/501100004047Karolinska Institutet; a national research infrastructure funded by the 10.13039/501100004359Swedish Research Council (dr.nr.2021-00179) and 10.13039/501100009252SciLifeLab.

The computations were enabled by resources provided by the National Academic Infrastructure for Supercomputing in Sweden (NAISS), partially funded by the Swedish Research Council through grant agreement no. 2022–06725, resources provided by Uppsala University at UPPMAX, and the Berzelius resource provided by the Knut and Alice Wallenberg Foundation at the National Supercomputer Center.

Work with SARS-CoV-2 was performed at the BSL-3 Biomedicum Core Facility, Karolinska Institute. We thank Oscar de Capetillo’s research group for kindly providing us with the A549^ACE2^ cell line, as well as Charlotte Stadler and for donating cell lines. We thank Christa Ringers and Anders Larsson for their contributions to data source management and dataset sharing. We thank Amelie Wenz for technical assistance with cell painting experiments and Martin Haraldsson and Annika Jenmalm Jensen for their chemical expertise.

## Author contributions

Conceptualization, P.O., O.S., B.S.-L., and J.C.-P.; methodology, E.A., J.R., M.T., H.A., D.N., A.K., P.G., and B.S.-L; investigation, E.A., J.R., M.T., H.A., D.N., A.K., and P.O.; writing – original draft, E.A and J.R.; writing – review and editing, P.O., O.S., and B.S.-L.; validation, E.A., J.R., and M.T.; formal analysis, E.A., J.R., M.T., H.A., S.P., M.L., and B.S.-L.; critical data review, A.S., M.d.K., T.A., A.Z., M.K., P.G., and D.L.; analysis and interpretation, J.C.-P. and O.S.; supervision, M.T., H.A., J.C.-P., B.S.-L., O.S., and P.O.; project administration, E.A and M.T.; funding acquisition, P.O., O.S., B.S.L., and J.P.C.

## Declaration of interests

J.C.-P. and O.S. declare ownership in Phenaros Pharmaceuticals.

## Declaration of generative AI and AI-assisted technologies in the writing process

During the preparation of this work, the author(s) used Chat-GPT 4/5 to reduce the word count, for grammar review, and proofreading. After using this tool or service, the author(s) reviewed and edited the content as needed and take full responsibility for the content of the publication.

## STAR★Methods

### Key resources table

**Table:** 

REAGENT or RESOURCE	SOURCE	IDENTIFIER
**Antibodies**		

Mouse monoclonal anti-spike antibody	GeneTex	Ref. GTX632604; RRID: AB_2864418
Mouse monoclonal anti-nucleocapsid protein antibody	Invitrogen	Ref. B46F; RRID: AB_1018422
Goat anti-mouse IgG	Invitrogen	Ref. A-21235 & A-21424; RRID: AB_2535804 & AB_141780
Goat anti-chicken IgG	Invitrogen	Ref. A-21449; RRID: AB_2535866

**Bacterial and virus strains**		

CoV-2/human/SWE/01/2020	Lentini et al.^61^	GenBank: MT093571.1
HCoV-229E	ATCC	Ref. VR-740
Zika virus	European Virus Archive	Ref. Strain H/PF/2013

**Chemicals, peptides, and recombinant proteins**		

SPECs drug repurposing library	https://www.specs.net/	N/A
CellTiter-Glo	Promega	Ref. G9243
Paraformaldehyde	Thermo Scientific	Ref. 28908
BSA	Sigma	Ref. A8022
Hoechst 33342	Invitrogen	Ref. H3570
SYTO 14	Invitrogen	Ref. S7576
Concanavalin A	Invitrogen	Ref. C11252
Phalloidin	Invitrogen	Ref. 10135092
CellTracker Deep Red Dye	Invitrogen	Ref. C34565
HCS LipidTOX Green Phospholipidosis Detection Reagent	Invitrogen	Ref. H34350

**Deposited data**		

Morphological features, code and data repository computing morphology to uninfected score and image analysis pipelines	This paper; GitHub	GitHub: https://github.com/pharmbio/sarscov2-phenomics-repurposing
Images	This paper; BioImage Archive	BioImage Archive: https://www.ebi.ac.uk/biostudies/bioimages/studies/S-BIAD2580; accession number: S-BIAD2580
Compound annotation of the entire drug repurposing library	Corsello et al.^29^	https://repo-hub.broadinstitute.org/repurposing#home
Compound annotation for the 74 hits	Reinshagen et al.^31^	https://doi.org/10.5281/zenodo.16359229

**Experimental models: Cell lines**		

Vero E6 (*C. sabaeus*)	Donated by cell profiling unit Charlotte Stadler	N/A
Calu-3 (*H. sapiens*)	ATCC	Ref. HTB-55
U2OS (*H. sapiens*)	Donated by cell profiling unit Charlotte Stadler	N/A
HepG2 (*H. sapiens*)	Donated by cell profiling unit Charlotte Stadler	N/A
Caco-2 (*H. sapiens*)	Donated by Jordi Carreras Puigvert	N/A
A549^ACE2^ (*H. sapiens*)	Donated by Oscar Fernandez-Capetillo lab	Porebski et al.^62^
U87-MG (*H. sapiens*)	ECACC	Ref. 890814
Vero (*C. sabaeus*)	ATCC	Ref. CCL-81
MRC-5 (*H. sapiens*)	ATCC	Ref. CCL-171

**Software and algorithms**		

Breeze	Potdar et al.^63^	https://breeze.fimm.fi/v2/
CellProfiler (v.4.2.1)	Stirling et al.^64^	https://cellprofiler.org/
CellPose (v2.0)	Stringer et al.^65^	https://www.cellpose.org/
R	R Core Team	http://cran.r-project.org/
R package *clusterProfiler* (v4.18.4))	Yu et al.^66^	https://bioconductor.org/packages/release/bioc/html/clusterProfiler.html
R package *aPEAR* (v1.0)	Kerseviciute I, Gordevicius J.^67^	https://github.com/kerseviciute/aPEAR
R package drc (v3.0-1)	Ritz C et al.^68^	https://cran.r-project.org/web/packages/drc/index.html
Python 3	Python Software Foundation	https://www.python.org/
GraphPad Prism (v.10.5.0 and v.11.0.0)	https://www.graphpad.com/	https://www.graphpad.com/
Harmony	Revvity	https://www.revvity.com/

**Other**		

Celphia Squid microscope	Cephla	https://www.cephla.com/
Image Xpress Micro XLS	Molecular Devices	https://www.moleculardevices.com/
Operetta	Revvity	https://www.revvity.com/

### Experimental model and study participant details

#### Cell lines and culture

African green monkey kidney cell line Vero E6 (African green monkey, female) was maintained in Dulbecco’s modified Eagle medium (DMEM) high glucose (Gibco) and supplemented with 1% L-glutamine (Cytiva). Validation experiments were performed using the human lung alveolar cell line A549^ACE2^ (human, male), which was genetically transduced to constitutively overexpress the ACE2 receptor, developed and donated by the Oscar Fernandez-Capetillo lab.^62^ The cells were maintained in DMEM/F-12 (Fisher Scientific) with GlutaMAX supplement and 1x non-essential amino acids (Cytiva). Cell lines used in the viability screen included Calu-3 (HTB-55, human, male) and Caco-2 (human, male), maintained in MEM with Earle’s salts (Sigma) supplemented with non-essential amino acids (Cytiva); U2OS (human, female), cultured in McCoy’s 5A medium (Sigma); and HepG2 (human, male), maintained in high-glucose DMEM. All cell lines were additionally supplemented with 10% fetal bovine serum (Gibco) and 1% penicillin/streptomycin (Cytiva).

Additional experiments with Vero cells (CCL-81, African green monkey, female) and U-87 MG cells (ref #890814, human) were maintained in Dulbecco′s Modified Eagle′s Medium (GIBCO), and MRC-5 cells (CCL-171, human, male) were cultured in Minimum Essential Media. These were supplemented with 10% FBS, 1% PS.

All cells were kept at 37°C in a humidified atmosphere with 5% CO_2_. They were detached using TrypLE Express (Gibco), counted, and diluted to appropriate densities with their respective media. Prior to experiments, cells were tested for mycoplasma using a luminescence-based assay (Lonza), and the cell lines were verified using STR profiling, except for the U2OS cell line, which was not profiled.

#### Biosafety

Experiments involving infectious viruses were performed under biosafety laboratory conditions in accordance with the Swedish Work and Health Authorities. Work with SARS-CoV-2 was conducted under BSL-3 conditions and work involving HCoV-229E and Zika virus was conducted under BSL-2 conditions.

#### Virus infection

The SARS-CoV-2 strain (CoV-2/human/SWE/01/2020; GenBank: MT093571.1) was obtained from the Public Health Agency of Sweden and propagated in Vero E6 cells and passaged three times. Viral titers were determined by plaque assay, and prepared stocks were stored at −80°C. Cells were infected in suspension containing serum-free media at an MOI of 0.05 for 1 h at 37°C and 5% CO_2_ atmosphere. Following infection, the cells were centrifuged at 1000 rpm for 5 min, washed with PBS, centrifuged, and resuspended in complete cell culture medium. The cells were seeded at an appropriate cell density in pre-spotted 384-well compound plates. Each plate included uninfected mock-control wells for normalization. The plates were incubated at 37°C in a 5% CO_2_ atmosphere for 24 h or 48 h, depending on the assay set up.

Zika virus (ZIKV, Strain H/PF/2013) was propagated in C6/36 cells (CRL-1660, Aedes albopictus, male) and viral titers determined in Vero cells. HCoV-229E (ATCC) were propagated and virus concentration determined in Huh-7 cells (human, male). Viral titers were determined by endpoint dilution assay and immunofluorescence imaging of a virus-specific protein. Cells were infected with ZIKV at an MOI 1 and incubated for 48h, and cells were infected with HCoV-229E at MOI 10 for 24h.

### Method details

#### Compound library and compound spotting

The drug repurposing library was sourced from SPECS in DMSO at 10 mM and reflects the set from the Broad Institute.^29^ Mechanism-of-action annotations for the full library were obtained from the Clue.io Repurposing Hub unless otherwise noted.^29^ Compound handling was performed by the SciLifeLab Compound Center (CBCS, Solna, Sweden). In brief, chemicals were dispensed between 2.5 nL and 40 nL using the Echo 55/6500 (Labcyte) liquid handler into 384-well assay plates (Phenoplate, Revvity) and stored at −20°C prior to experimentation. For the full repurposing library (*n* = 5275), compounds were distributed across the plates with one technical replicate and two biological replicates. The validation screen by Cell Painting in A549^ACE2^ was performed in a 4-point-dose-response with concentrations ranging from 0.1 to 30 μM, in three replicates placed on separate multiwell plates. To reduce bias by positional effects in the microwell plates, the conditions were distributed over the plates using Plate Layouts using Artificial Intelligence Design.^40^

#### Viability screen

The viability screen was conducted using five cell lines: Vero E6, Calu-3, U2OS, HepG2, and Caco-2; and were tested in five-point dose response ranging from 1 nM to 10 μM. Cells were seeded at 25 μL per well to pre-spotted 384-well white cell culture plates (Greiner Cellstar) using Multidrop Combi (Thermo Scientific) and were incubated at standard conditions for approximately 72 h. The plates were placed in humidity boxes to maintain optimal conditions during incubation. Cell viability was assessed using the CellTiter-Glo 2.0 assay (Promega), following the manufacturer’s protocol and luminescence was measured using the Enspire reader (PerkinElmer). The data were analyzed using the web-based software Breeze with Drug Sensitivity Score (DSS) as one of the readouts, a metric derived from dose-response curves.^63^^,^^69^ A DSS of zero indicates no toxicity, while a DSS >10 indicates compound toxicity.

#### Antiviral rescue of cytopathic effect (CPE)

Infected Vero E6 cells were seeded at a density of 4000 cells per well in 30 μL using a Viaflo 384 (Integra Biosciences) and incubated under standard conditions. Compounds were tested in five doses, ranging from 0.001 to 10 μM, with uninfected and infected controls. At 48 h post-infection (hpi), cell viability was measured by luminescence using the SpectraMax iD5 (Molecular Devices) and the CellTiter-Glo assay 2.0 (Promega), following the manufacturer’s protocol. The data were normalized to uninfected and infected DMSO control wells, and inhibition of cytopathicity (%) was calculated relative to these controls. A compound was defined as a hit if any of the tested doses resulted in an antiviral efficacy exceeding 15%, defined by mean plus three standard deviations of all compound-treated wells.

#### Morphological profiling and immunostaining

Infected Vero E6 cells (2500/well, 30 μL), and A549^ACE2^ cells (4000 cells/well, 30 μL) were seeded using a Viaflo 384 onto pre-spotted 384-well assay plates (Phenoplate, Revvity). The plates were then incubated for 24 h, after which they were fixed with 4% paraformaldehyde (Thermo Scientific), washed twice with PBS, and stained using our adapted Cell Painting assay.^27^ The cells were permeabilized with Triton X-(0.1% Triton X-100), blocked with BSA (0.2%, Sigma) and FBS (9%, Gibco), and incubated overnight with primary antibodies: anti-spike (GeneTexA) for Vero E6 and anti-nucleocapsid (Invitrogen) for A549^ACE2^. Then plates were washed 3x with PBS, followed by staining with a dye mix including Hoechst, SYTO 14, Concanavalin A, WGA, Phalloidin, and a secondary antibody (Invitrogen). Plates were washed an additional 3x with PBS and then stored at 4°C until imaged. Liquid handling was automated using an automated reagent dispenser (Biotek Multiflo FX).

Fluorescent images were acquired using an Image Xpress Micro XLS (Molecular Devices) microscope with a 20X objective using laser-based autofocus for Vero E6 experiments, or with a Squid (Cephla) microscope for A549^ACE2^ cells. In total, 9 sites per well were captured using 5 fluorescence channels. The images were analyzed with the open-source image analysis software CellProfiler version 4.2.1.^64^ The Cellpose v2.0 cyto2 model was used for segmentation of nuclei and cells.^65^ A quality control pipeline and illumination correction pipeline was used to remove corrupted images. A total of 2122 features per object were extracted using CellProfiler’s AreaShape, Correlation, Intensity, Granularity, Location, Neighbors, and RadialDistribution modules.

Downstream analysis of the feature data was performed using Python 3, using Jupyter notebooks. Images flagged as outliers based on a threshold of ±10 standard deviations from the mean were excluded. Mean feature values were computed on the well level. Invariant features (standard deviation ≤0.0001) or features with extreme variation (>15 standard deviations), and features with missing data were removed. MAD normalization was applied by subtracting the median and dividing by the MAD of the corresponding DMSO samples on each plate. To reduce the influence of extreme values, all feature values were clipped to the range of −50 to 50.

PLS regression was used to model the phenotypic separation between infected and uninfected controls, where uninfected wells were labeled as 1 and infected wells as 0. Predictive power (*Q*^2^) and accuracy (*R*^2^) were calculated for the PLS-DA model, which were estimated by 3-fold cross-validation. For each compound, the mean prediction score was calculated reflecting the extent to which the compound’s phenotypic profile resembled the uninfected state. In parallel, we calculated the Pearson correlation coefficient between each compound’s profile and the mean profile of the uninfected controls. To quantify overall similarity to the uninfected phenotype, we averaged the PLS-DA prediction score and the correlation coefficient, yielding a morphology-to-uninfected-control score (referred to as *morphology score*) for each compound. Processing of features for the analysis of non-infected cell populations was performed using Pycytominer. Features were classified using the antibody channel, and infected and non-infected cells were aggregated at the well level, annotated with metadata, and normalized against controls (DMSO) using the mad_robustize method. Blocklisted, correlated, outlier, invariant, noisy, and NA-heavy features were removed, resulting in 963 features. Consensus profiles were then generated at the compound–dose level using the MODZ function.^70^

For visualization of the affected features in radar plots, the absolute mean of the mad-normalized features was computed, grouped by CellProfiler module (Intensity, Correlation, Granularity, Location, RadialDistribution), and stain. Area-shape related features were grouped by cell compartment, i.e., Cell (C), Cytoplasm (Cy) and Nucleus (N). Neighboring related features were grouped by Cell (C) and Nucleus (N).

The antibody staining included in the assay, was used to quantify the number of infected cells per well. A cell was considered infected if the virus-specific antibody intensity exceeded a threshold defined as the mean plus three standard deviations of the antibody signal in the blank (uninfected) control wells. The set-off was determined on a plate-to-plate basis to avoid bias by batch effects and was checked for accuracy by checking for false positives in non-infected wells.

#### Drug-induced phospholipidosis (DIPL) evaluation

Compounds were dispensed in 384-well plates as previously described, in six 3-fold dilutions (0.3–30 μM) and in technical duplicates. Uninfected A549^ACE2^ cells were split, spun down and resuspended in serum-free, phenol red-free DMEM/F-12 (Gibco) medium. The cells were incubated with CellTracker Deep Red Dye (13 μM, Thermo Fisher Scientific) for 40 min, centrifuged and resuspended with serum-containing phenol red-free DMEM/F-12 medium. Hoechst 33342 (120 nM, Invitrogen) and HCS LipidTOX Green Phospholipidosis Detection Reagent (0.25X, Thermo Fisher Scientific) were added to the cell suspension. The cells were seeded using multidrop (Thermo Fisher Scientific) at a density of 1500 cells per well (30 μL) and were maintained at standard conditions. The plates were imaged live with the Operetta microscope (Revvity), using 20X confocal water objective, at 24 h, 48 h, and 72 h post-seeding. The images from the Operetta were subsequently analyzed using the Harmony software (Revvity, USA).

An analysis pipeline for detecting and quantifying phospholipidosis was established based on the average area of spots per cell, detected by the LipidTOX dye. Assay quality (0.3% DMSO as negative control and 10 μM tamoxifen as a positive control) was calculated according to standard procedures and plates above Z′-factor threshold of 0.4 were considered for the experiment.^71^ Defining the non-DIPL inducers, a primary threshold of 9% DIPL was calculated as mean plus three standard deviations of the DMSO control. A secondary threshold of 20% DIPL was set to distinguish the low from moderate-to-high DIPL inducers. Wells with low cell count were excluded from the analysis and were defined as <100 cells/well at 24h, <200 cells/well at 48h, and <300 cells/well at 72h. Dose-response curves were generated for compounds with at least four doses and a minimum of DIPL at 50%. Compounds that did not meet this criterion were excluded from the curve fitting due to insufficient doses or DIPL response.

Compounds were fitted to a four-parameter logistic (LL.4) model to create dose-response curves, and the curve fitting was performed in R (version 2024.12.0 + 467) using the drc (v3.0-1) package.

#### Gene ontology enrichment analysis

For the 74 candidate compounds, the annotations from Clue.io Repurposing Hub^29^ were complemented with compound targets systematically collected from ChEMBL bioactivity data, now referred to as REMEDi4ALL annotations.^31^^,^^72^ The following parameters were set for the REMEDi4ALL target list from: *Homo sapiens* proteins with a confidence score ≥8 and pChEMBL score ≥6 from binding and functional assays were included. This expanded the target list from 150 to 700 targets.

The GO analysis was conducted using the *enrichGO()* function from the *clusterProfiler* R package (v4.18.4), focusing on the Biological Process ontology and the human gene annotation database *org.Hs.eg.db*. The following parameters were applied: *p*-value adjustment by the Benjamini-Hochberg method, *p*-value cutoff of 0.05, and a q-value cutoff of 0.2. The enrichment was visualized using the top 50 GO terms using *enrichmentNetwork()* function from the Advanced Pathway Enrichment Analysis Representation (*aPEAR)* package (v1.0).^73^

#### Dose-response evaluation by immunostaining of ten prioritized compounds

Infection of A549^ACE2^ cells and subsequent cell seeding, fixation and permeabilization is described in the STAR Methods section: morphological profiling and immunostaining. Plates were imaged in Opera Phoenix using non-confocal objective and 10x resolution. Image analysis was conducted in the Harmony (Revvity, USA) software to quantify infected cells from the SARS-CoV-2 nucleocapsid protein signal. Wells were normalized to DMSO controls, and the proportion of infected cells were calculated as the number of infected cells per well divided by the total number of nuclei, based on the Hoechst signal. Data points were fitted using a four-parameter logistic model in GraphPad Prism (v.11.0.0).

### Quantification and statistical analysis

Thresholds for infection classification, hit selection in the CPE assay, and primary classification of drug-induced phospholipidosis were defined as the mean plus three standard deviations of the corresponding control population. Additional assay-specific thresholds are described in the relevant STAR Methods sections. For analysis of morphological data, PLS-DA model performance was evaluated using Q2 and R2 values. Morphological similarity to the uninfected phenotype was quantified using the average of the PLS-DA prediction score and the Pearson correlation coefficient between each compound profile and the mean profile of the uninfected controls. Additional details including replicate numbers, quality control metrics, and assay-specific analysis are provided in the corresponding Methods text and/or figure legends. Python 3 was used for analysis of morphological data, R and GraphPad Prism used for dose-response curve fitting and when stated in the text.

## Supplemental information

Document S1. Figures S1–S4

Table S1. Viability screen results in Calu3 cells with the full drug repurposing library in five doses, related to Figure 2

Table S2. Viability screen results in HepG2 cells with the full drug repurposing library in five doses, related to Figure 2

Table S3. Viability screen results in U2OS cells with the full drug repurposing library in five doses, related to Figure 2

Table S4. Viability screen results in Vero E6 cells with the full drug repurposing library in five doses, related to Figure 2

Table S5. Viability screen results in Caco2 cells with the full drug repurposing library in five doses, related to Figure 2

Table S6. Primary screen results from cytopathic effect assay in Vero E6 cells and SARS-CoV-2 (MOI 0.05), related to Figures 2 and S1 The full drug repurposing library was screened in 5 doses.

Table S7. Primary screen results from the cell painting assay in Vero E6 cells and SARS-CoV-2 (MOI 0.05), related to Figures 2 and S1 Morphology score readout from cell painting and SARS-CoV-2 antibody readout to measure infection rate. The full drug repurposing library was screened at a single dose.

Table S8. Validation screen results from the cell painting assay in A549^ACE2^ cells and SARS-CoV-2 (MOI 0.05), related to Figure 3 Morphology score readout from cell painting and SARS-CoV-2 antibody readout to measure infection rate. A subset of the drug repurposing library was screened (*n* = 324) in 4 doses.

Table S9. Counter screen for drug-induced phospholipidosis (DIPL) in non-infected A549^ACE2^ cells, related to Figures 3 and S2 A subset of the drug repurposing library (*n* = 324) in 6 doses. Immunofluorescent readout in live cells at 24, 48, and 72 h.

Table S10. 74 validated hits, related to Figure 4 Prioritized list and targets with metadata.
