# Supplementary material for: A host-centric morphological profiling approach to identify repurposed antiviral drugs
Source: iScience. 2026 Jul 10;29(8):116673. doi: 10.1016/j.isci.2026.116673 (PMC13380429; doi:10.1016/j.isci.2026.116673)
Supplement: Document S1. Figures S1–S4 [file mmc1.pdf]

iScience, Volume *29*

Supplemental information

A host-centric morphological profiling approach to identify repurposed antiviral drugs

Elin Asp, Jonne Rietdijk, Marianna Tampere, Hanna Axelsson, Duncan Njenda, Swapnil Potdar, Adelinn Kalman, Polina Georgieva, Maris Lapins, Flavio Ballante, Alicia Soler, Martin de Kort, Tero Aittokallio, Andrea Zaliani, Maria Kuzikov, Philip Gribbon, Donald Lo, Jordi Carreras-Puigvert, Brinton Seashore-Ludlow, Ola Spjuth, and Päivi Östling

Supplementary Figures

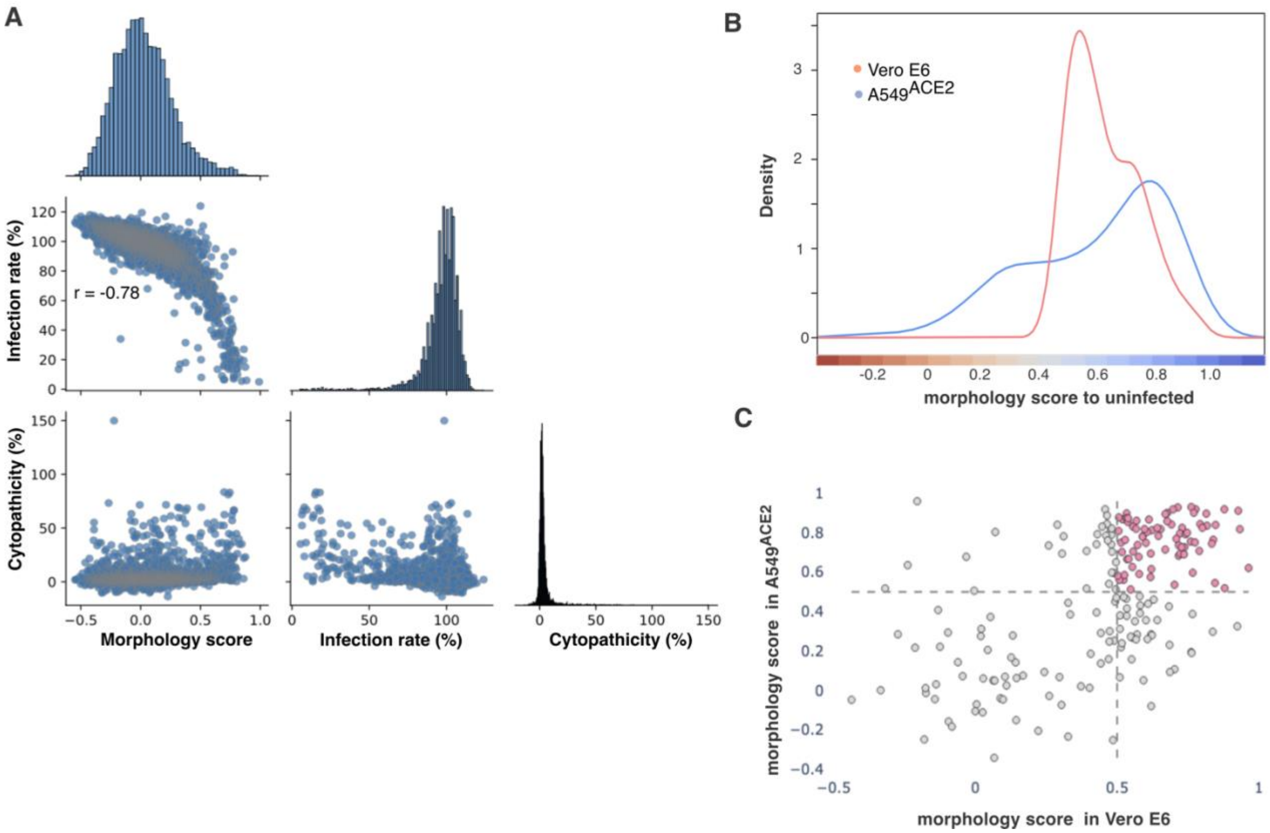

Supplementary Figure S1. Comparison of primary and validation screening results; related to Figure 2 and 3. A. Pairwise plots of morphology score, inhibition of cytopathicity (%), and inhibition of viral infection for each perturbation (compound–dose combination). **B.** Density plots showing the distribution of morphology scores for the 150 prioritized compounds in Vero E6 and their respective scores in the $A549^{ACE2}$ cell line. **C.** Scatterplot comparing morphology scores between Vero-E6 and $A549^{ACE2}$ cells at matched doses for all 324 prioritized compounds. In pink are highlighted the compounds that achieved a morphology score of >0.5 in both cell models.

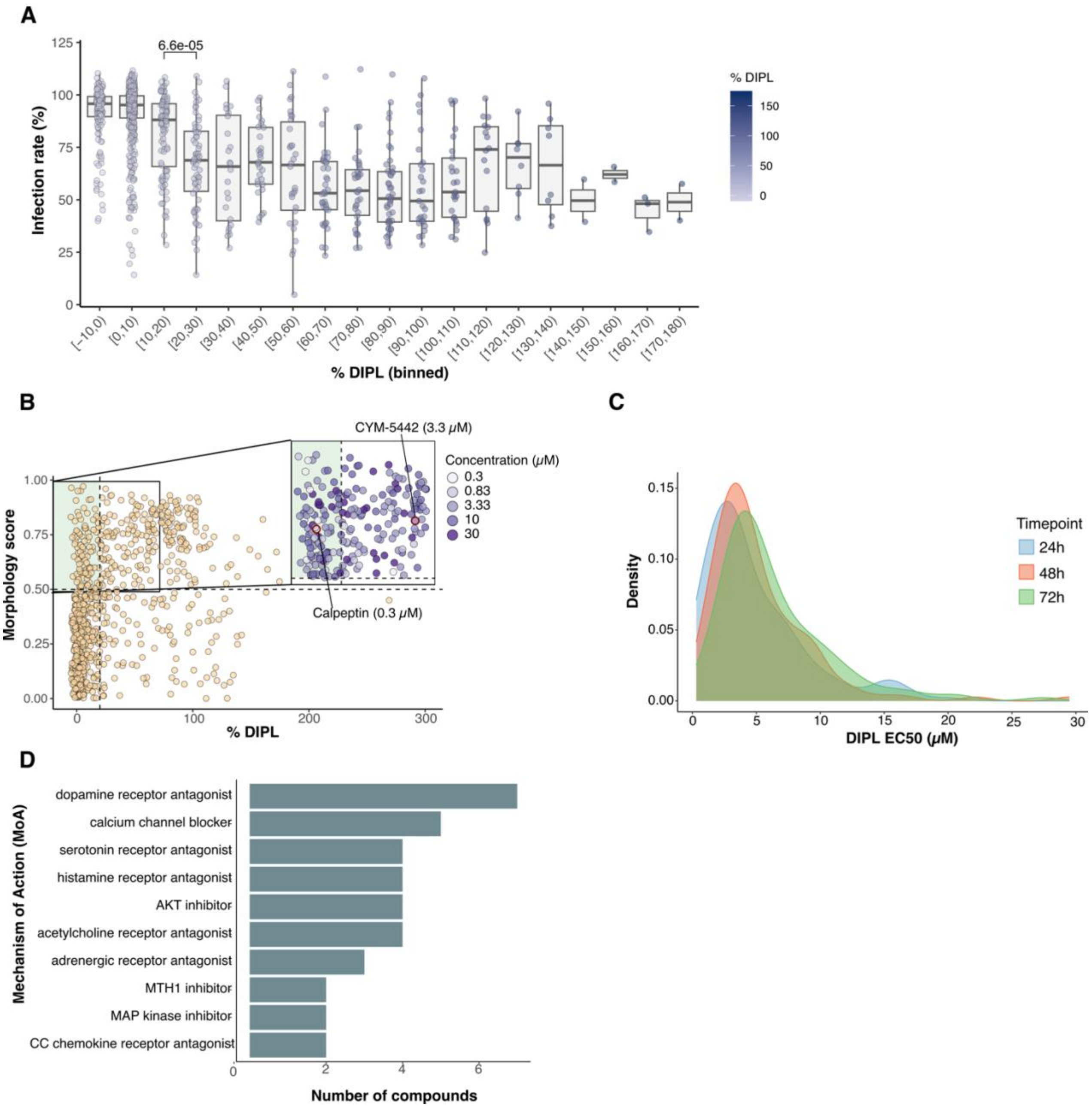

Supplementary Figure S2. DIPL evaluation across timepoints and relationship with SARS-CoV-2 infection; related to figure 3. A. SARS-CoV-2 infection rate across binned % DIPL values. Compounds were grouped into DIPL bins (10%-width), and infection rates are displayed as boxplots with individual data points overlaid. A significant decrease in infection rate was observed between 10-20% and 20-30% DIPL bins (Wilcoxon rank sum test, $p = 6.6 \times 10^{-5}$). **B.** A scatter plot showing the relationship between DIPL (x-axis, % normalized signal) and morphology score (y-axis) across tested concentrations of the 324 hit compounds. Each point represents a compound-concentration pair. Cut-offs show moderate DIPL level (<20%) and morphology score >0.5 for non-infected morphology. Compounds within the thresholds are indicated by a green-shaded area (n = 74). A close-up highlights compounds with non-infected morphology

with varying DIPL levels, colored by concentration. Two compounds, CYM-5442 (3.3 µM) and Calpeptin (0.3 µM) are highlighted. **C.** Density plot showing EC_{50} values for DIPL across 24h, 48h, and 72h. **D.** The top ten most frequent mechanisms of action among DIPL inducers.

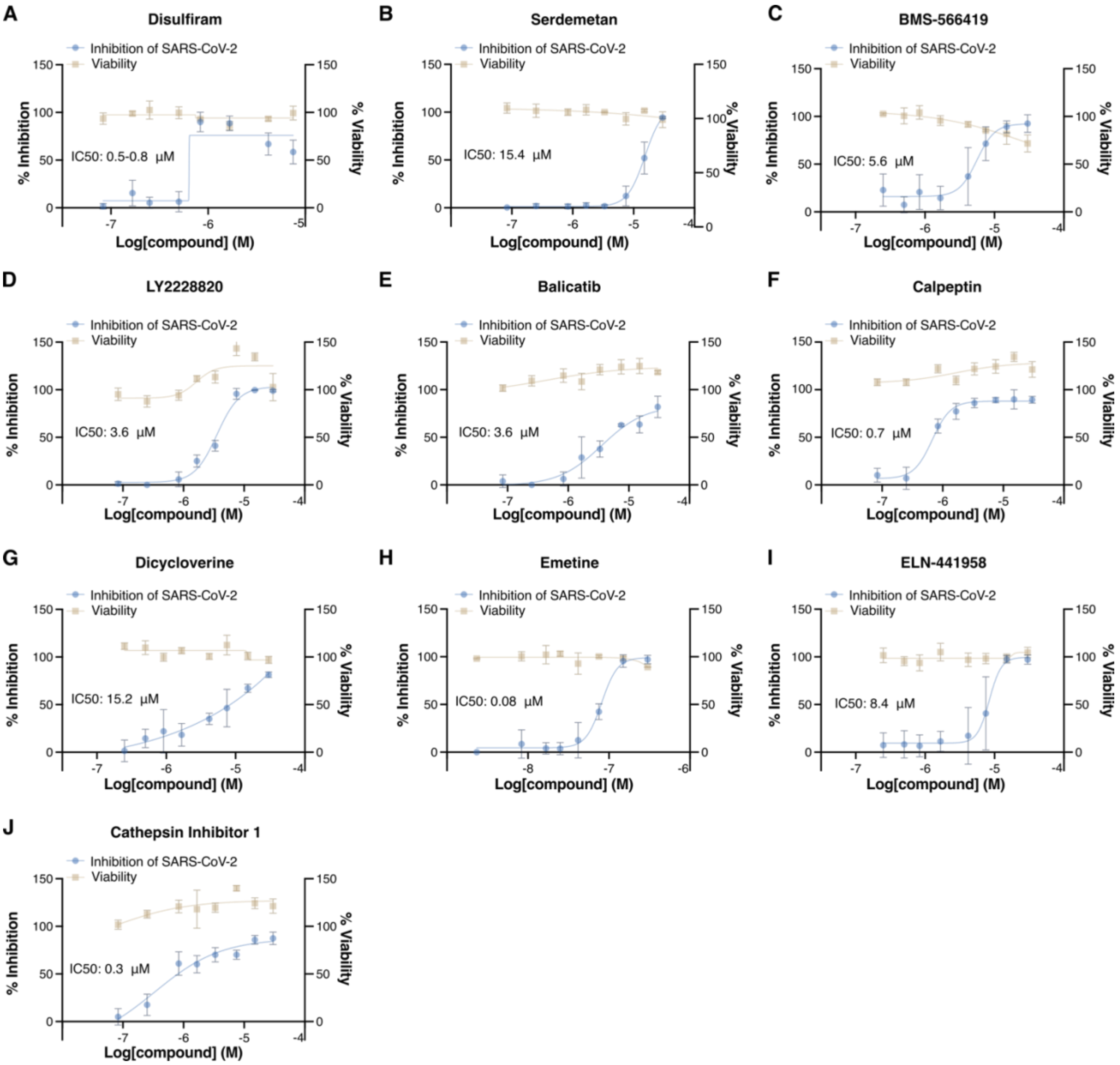

Supplementary Figure S3. Dose-response validation of the top ten ranked compounds; related to Figure 4. A-J. A549[ACE2] cells were infected with SARS-CoV-2 at MOI 0.05 and seeded onto compound-treated wells. At 24 h post-infection, cells were fixed and stained for immunofluorescence imaging. Infection was quantified based on SARS-CoV-2 nucleocapsid protein levels and normalized to DMSO-treated wells. The left y-axis shows the percentage inhibition of SARS-CoV-2 infection, and the right y-axis shows percentage cell viability. Data points represent the mean ± SD of three replicates. Dose-response

curves were generated using a four-parameter logistic model in GraphPad Prism (v.11.0.0). IC_{50}-values were derived from the fitted curves with exception of disulfiram, for which had an unstable fit and its IC_{50}-value was estimated based on the concentration spanning 50% inhibition.

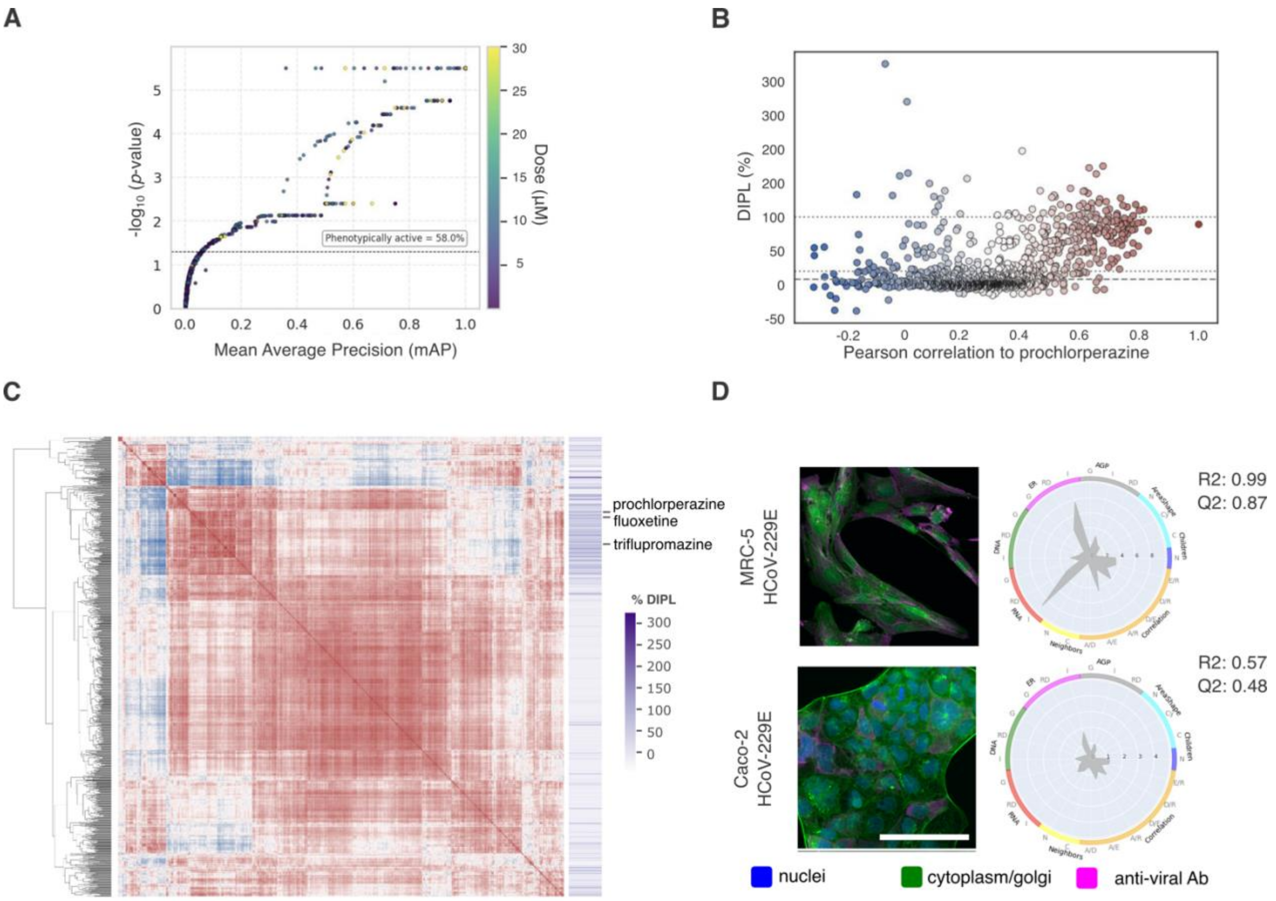

Supplementary Figure S4. Leveraging morphological profiling; related to Figure 5. A. Mean average precision and

$-\log_{10}$(p-value) for morphological profiles of uninfected cells. The dashed line indicates the significance threshold. The percentage of significantly changed morphological profiles is annotated above and data points are color-coded by compound concentration. **B.** Pearson correlation to fluoxetine hydrochloride versus percentage of phospholipidosis induction across compounds. **C**. Clustermap of Pearson correlations between compound-dose morphological profiles in uninfected cells. Highlighted compounds include prochlorperazine, triflupromazine, and fluoxetine. DIPL% values are represented on a continuous color bar. **D.** Representative Cell Painting images and radar plots for virus-infected cells in two cell line-virus combinations: MRC-5 with HCoV-229E, and Caco-2 with HCoV-229E. Profile strength is summarized using PLS-DA R^2 and Q^2 evaluation metrics. Fluorescence channels indicate nuclei (blue), virus-specific antibody (fuchsia), and cytoskeleton, Golgi, and plasma membrane (green). Scale bar = 100 µm.
